# A Previously Unrecognized Molecular Landscape of Lynch Syndrome in the Mexican Population

**DOI:** 10.3390/ijms231911549

**Published:** 2022-09-30

**Authors:** Alejandra Padua-Bracho, José A. Velázquez-Aragón, Verónica Fragoso-Ontiveros, Paulina María Nuñez-Martínez, María de la Luz Mejía Aguayo, Yuliana Sánchez-Contreras, Miguel Angel Ramirez-Otero, Marcela Angélica De la Fuente-Hernández, Silvia Vidal-Millán, Talia Wegman-Ostrosky, Abraham Pedroza-Torres, Cristian Arriaga-Canon, Luis A. Herrera-Montalvo, Rosa Maria Alvarez-Gómez

**Affiliations:** 1 Hereditary Cancer Clinic, National Cancer Institute, Mexico City 14080, Mexico; 2 Postgraduate Program Doctorate in Biomedicine and Molecular Biotechnology, National Polytechnic Institute, Mexico City 11340, Mexico; 3 Experimental Oncology Laboratory, National Pediatric Institute, Mexico City 04530, Mexico; 4 Basic Research Sub Direction, National Cancer Institute, Mexico City 14080, Mexico; 5 General Management, National Institute of Genomic Medicine, Mexico City 14610, Mexico

**Keywords:** Lynch syndrome, pathogenic variants, mutations, variants of uncertain significance, MSH2, MLH1, MSH6, EPCAM, PMS2

## Abstract

Lynch syndrome (LS) is the main hereditary colorectal cancer syndrome. There have been few reports regarding the clinical and molecular characteristics of LS patients in Latin America; this is particularly true in the Mexican population, where no information is available. The present study aims to describe the clinical and molecular spectrum of variants in a cohort of patients diagnosed with LS in Mexico. We present a retrospective analysis of 412 patients with suspected LS, whose main site of cancer diagnosis was the colon (58.25%), followed by the endometrium (18.93%). Next-generation sequencing analysis, with an extensive multigene panel, showed that 27.1% (112/414) had a variant in one of the genes of the mismatch repair pathway (MMR); 30.4% (126/414) had a variant in non-MMR genes such as *CHEK2*, *APC*, *MUTYH*, *BRCA1*, and *BRCA2*; and 42.5% (176/414) had no genetic variants. Most of the variants were found in *MLH1*. Pathogenic variants (PVs) in MMR genes were identified in 65.7% (96/146) of the total PVs, and 34.24% (45/146) were in non-MMR genes. Molecular and clinical characterization of patients with LS in specific populations allowed personalized follow-up, with the option for targeted treatment with immune checkpoint inhibitors and the development of public health policies. Moreover, such characterization allows for family cascade testing and consequent prevention strategies.

## 1. Introduction

Lynch syndrome (LS) is the most common hereditary syndrome of colorectal cancer. It is also associated with predisposition to several extracolonic neoplasms, with endometrial carcinoma being the most common, and an increased risk of cancer of the ovary, small intestine, stomach, urinary tract, pancreas, and brain [1]. It is an autosomal dominant syndrome caused by germline mutations in one of the genes of the mismatch repair (MMR) pathway: *MLH1*, *MSH2*, *MSH6*, and *PMS2* [2,3]. The germline pathogenic variant (PV), combined with a second somatic acquired pathogenic variant in the wildtype allele, results in complete loss of the MMR pathway function in affected cells [4], resulting in an inability to repair mismatch errors and microsatellite instability (MSI), which is present in 95% of all tumors associated with LS [4,5]. Part of the process of identifying individuals at risk of LS relies on the review of the individual and family history of cancer, where the Amsterdam and Bethesda criteria are evaluated [3]. Two types of tests are used to screen for LS in tumor samples: microsatellite instability (MSI) and immunohistochemistry (IHC) for the MMR proteins. When MSI is high (MSI-H) or there is a loss of MMR proteins, germline DNA testing is recommended to confirm the LS diagnosis [6]. Diagnosis is relevant for risk-specific screening and follow-up for the associated neoplasms, as well as to determine potential treatment options, such as drugs targeting PD1 and CTLA-4 proteins [7,8,9,10]. It is also fundamental for the prevention of cancer in relatives at risk of LS [2].

The increased lifetime risk of developing a neoplasm of the syndrome spectrum appears to be dependent on the mutated MMR gene. For *MLH1,* the highest risk corresponds to colorectal cancer (46%), followed by endometrial (43%), gastrointestinal (21%), ovarian (10%), and urinary cancer (8%); for *MSH2,* the highest risk relates to endometrial cancer (57%), followed by colorectal (43%), ovarian (17%), urinary (25%), and gastrointestinal cancer (10%); *MSH6* displays risk of endometrial (46%), colorectal (15%), ovarian (13%), urinary (11%), and gastrointestinal cancer (7%) [11]. Lastly, *PMS2,* demonstrates a high risk of endometrial cancer (26%), followed by colon (20%), renal (3.7%), and ovarian cancer (3%) [12].

The spectrum of genetic variants of LS has been extensively studied in colorectal patients from North America, Europe, Australia, and Asia. In these studies, a higher prevalence of PV has been observed in patients with *MLH1* and *MSH2* [13,14]. The molecular and clinical characteristics, as well as the spectrum of MMR variants in LS, have been poorly studied in Latin America. According to reports collected in Argentina, Brazil, Colombia, Uruguay, Chile, Bolivia, Peru, Costa Rica, Puerto Rico, and Ecuador, the combined PV prevalence has been estimated to be between 43 and 54% for *MLH1*; 32.4 to 43% for *MSH2*; 9 to 10% for *MSH6*%; 3 to 10% for *PMS2*, and nearly 1% for *EPCAM* [13,15,16]. To the best of our knowledge, there are scarce reports of the genetic variants (MMR genes) identified in Mexican patients with LS [15,16]. 

Our research aims to describe the clinical and molecular characteristics identified through a comprehensive next-generation sequencing (NGS) multigene panel, in a cohort of LS patients diagnosed at the Hereditary Cancer Clinic, of the National Cancer Institute, Mexico.

## 2. Results

A total of 412 patients with suspected LS were recruited from 2016 to 2021, through the Hereditary Cancer Clinic of the National Cancer Institute in Mexico City, Mexico. Patients underwent a cancer risk assessment by medical geneticists, with the corresponding pre- and post-test genetic counseling. LS patients were selected using Amsterdam and Bethesda criteria, as well as the NCCN guidelines. For the purposes of describing our population, we grouped patients under the term “suspected Lynch syndrome” for those who met the clinical criteria and underwent genetic testing. Based on molecular analysis results, we further divided them into “MMR”, comprising those patients with pathogenic variants in MMR genes, and therefore with LS; “no variant identified”, for those patients with a negative result; and, “non-MMR”, comprising those patients with a variant in a gene other than an MMR gene.

Of the 412 patients, 71.84% (296/412) were female and 28.16% (116/412) were male. The mean age at diagnosis was 40.82 years, with a minimum age of 16 and maximum age of 78 years (SD 12.09). The mean current age was 44.27 years (SD 12.87). A positive cancer family history was present in 47.33% (195/412) of patients.

With respect to clinical stage, 14.81% (61/412) presented at stage IV; of these, no pathogenic variant was identified in 57.38% (35/61) of patients. Of the remaining stage IV patients, 32.79% (20/61) presented with a variant in genes that do not belong to the MMR genes, and 9.84% (6/61) had a variant in the MMR genes. Stage IIA patients accounted for 12.86% (53/412); of these, 42.28% (24/53) had a variant in the MMR genes, 33.96% (18/53) had no variants identified, and 20.75% (11/53) had a variant in non-MMR genes (Table 1 and Table S1).

Concerning tumor diagnosis associated with LS, 58.25% (240/412) corresponded to colorectal cancer, followed by endometrial (18.93%, 78/412), ovarian (10.44%, 10/412), breast (6.31%, 26/412), and gastric cancer (2.43%, 10/412). In addition, a few cases of cervical (0.73%, 3/412), pancreatic, and vaginal cancer (0.49% 2/412, each); and prostate, renal, skin, thyroid, brain, and appendix cancer were also reported (0.24%, 1/412 each) (Table 2). Regarding the status of patients at the end of the study, 62.62% (258/412) were alive without disease; most (101/412) of whom had a pathogenic variant that was not identified (Table 1).

**Figure 1:**
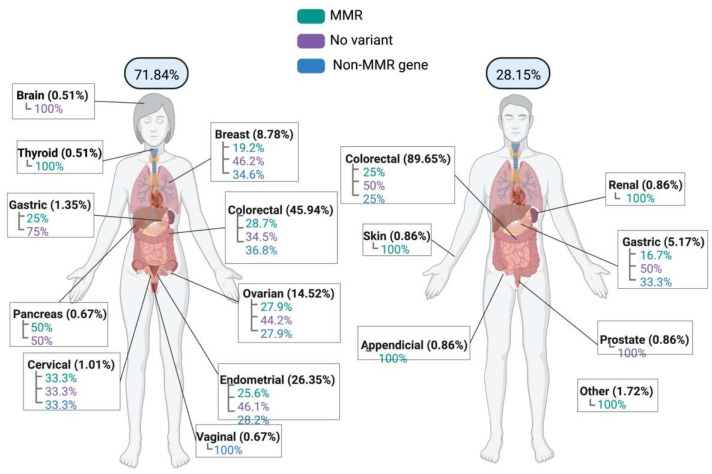
Incidence site in cases of suspected Lynch syndrome by sex. The figure shows the percentages of women and men with suspected Lynch syndrome in the cohort, as well as sites of the first tumor diagnosis reported in both women and men. The percentage of reported cases relative to the total number of women and men is shown in parentheses. In each incidence site box, the percentage of cases with variants in MMR (turquoise), no variant identified (purple), and variants in non-MMR genes (blue) are shown.

The distribution according to the first cancer diagnosis and sex in the patients studied is shown in Figure 1. In women, the most common site corresponded to colorectal cancer, in 45.94% of cases (136/296). Of these, 36.8% (50/136) had a variant in non-MMR genes, 34.5% (47/136) had no variant identified, and 28.7% (39/136) had variants in MMR genes. The second most common cancer was endometrial cancer, in 26.35% of cases (78/296), where 46.1% (36/78) corresponded to no variant being identified; 28.2% (22/78) to variants in non-MMR genes, and 25.6% (20/78) to variants in MMR genes. Ovarian cancer ranked third in the frequency of first cancer diagnosis, with 14.52% of cases (43/296), where 44.2% (19/34) corresponded to cases without an identified variant, and 27.9% had variants in non-MMR genes (12/43) and MMR genes (12/43). Fourth place corresponded to breast cancer, which was found in 8.78% of cases (26/296), where 46.2% (12/26) were without an identified variant, 34.6% (9/26) had a variant in non-MMR genes, and 19.2% (5/26) had variants in MMR genes. Gastric cancer was reported in 1.35% of cases (4/296), where 75% (3/4) correspond to cases with no variant identified, and 25% (1/4) had variants in MMR genes. The least representative occurrences were: pancreatic cancer and vaginal cancer at 0.67% (2/296 each), where one patient (50%) with pancreatic cancer had a variant in an MMR gene and the other had no variant identified, while for vaginal cancer, 100% of the two cases presented variants in non-MMR genes; and thyroid and brain cancer at 0.51% (1/296 each), where the only patient with thyroid cancer presented a variant in an MMR gene, while for brain cancer, no variant was identified.

For male patients, 89.65% (104/116) presented with colorectal cancer, where 50% (52/104) of cases had no variant identified, and 25% (26/104 each) had a variant in non-MMR genes and MMR genes. The second most common cancer was gastric cancer, found in 5.17% (6/116) of patients, where no variant was identified in 50% (3/6) of cases, 33.3% (2/6) had a variant in non-MMR genes, and 16.7% (1/6) had a variant in MMR genes. Prostate cancer was found in a smaller proportion of patients, 0.86% (1/116), where no variant was identified in one patient; along with skin, renal, and appendix cancer in 0.86% (1/116) of patients, where a variant in an MMR gene was identified in all three cases.

Regarding the disease subtype, in the cases of colorectal cancer, 95.42% (229/240) corresponded to adenocarcinoma, where the majority (93) of cases had no variant identified, 74 cases had a variant in non-MMR genes, and 62 cases had a variant in MMR genes. For endometrial cancer, 67.95% (53/78) corresponded to the endometrial adenocarcinoma subtype, for which 22.64% (12/53) had a variant in MMR genes. In the case of ovarian cancer, the subtype with the highest prevalence was endometrioid adenocarcinoma, at 37.21% (16/43), where five cases had a variant in MMR genes. Of the breast cancer cases, 80.77% (21/26) were invasive ductal carcinoma, and MMR gene variants were found in 23.81% (5/26) (Table 3).

**Figure 2:**
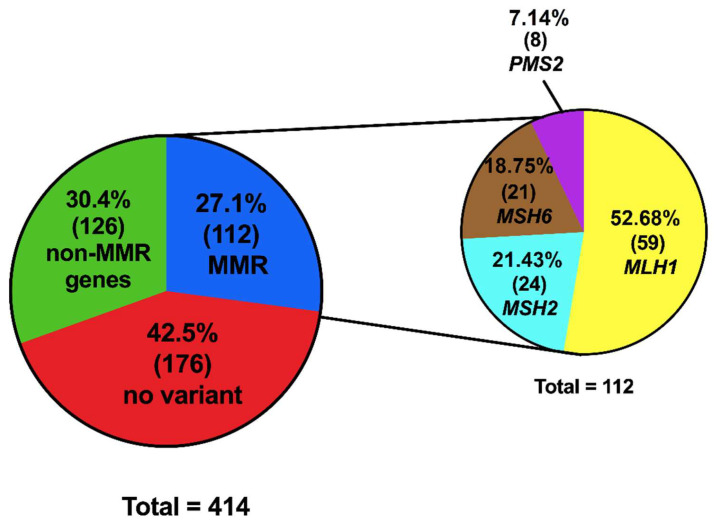
Variants identified in the cohort of patients. A total number of 414 variants were detected in 412 patients, two patients were double hetero-zygous for MMR. The figure shows the percentage of the results obtained in the multigene panel; additionally, the figure shows the percentage of variants identified in each of the MMR genes.

By analyzing the results of the multigene panel performed on the patients with LS, 42.5% (176/414) had no variant identified (Figure 2). To complement the molecular approach, because of important clinical LS suspicion, *MLH1* and *MSH2* multiplex ligation-dependent probe amplification (MLPA) was performed on 36 of the 412 patients (8.73%). The result was negative for all of them, since no deletions or duplications of exons of the explored genes were identified.

Of the patients with no variant identified, 67.43% (118/175) were women and 32.57% (57/175) were men; the mean age at diagnosis was 40.56 years, with a minimum age of 17 years and a maximum of 78 years. The mean current age was 40.656 years. A total of 57.06% (101/175) were alive without disease at the end of this study. Clinical stage IV disease was present in 19.77% (35/175) of patients. The remaining socio-demographic characteristics of this group are shown in Table 1.

In the total studied population, 27.1% (112/414patients) presented variants in one of the MMR genes; 52.68% (59/112) corresponded to *MLH1*, 21.43% (24/112) to *MSH2*, 18.75% (21/112) to *MSH6,* and 7.14% (8/112) to *PMS2* (Figure 2). The mean age at diagnosis in this subpopulation was 40.63 years, with a minimum age of 16 and maximum age of 66 years (SD 11.54). A positive cancer family history was present in 38.46% (75/412) of cases. Concerning patients’ status at the end of this study, 72.07% (80/111) were alive without disease, with an average current age of 45.18 years (SD 10.55). Clinical stage IIA was the most prevalent stage in 21.62% (24/111) of cases.

When a variant was identified in one of the MMR genes, the majority (58.56%, 65/111) corresponded to colorectal cancer, followed by endometrial cancer (18.02%, 20/111), ovarian cancer (10.81%, 12/111), breast cancer (4.50%, 5/111), and gastric cancer (1.80%, 2/111). In addition, a variant was identified for cervical, renal, skin, thyroid, and pancreatic cancers (Table 2).

**Figure 3:**
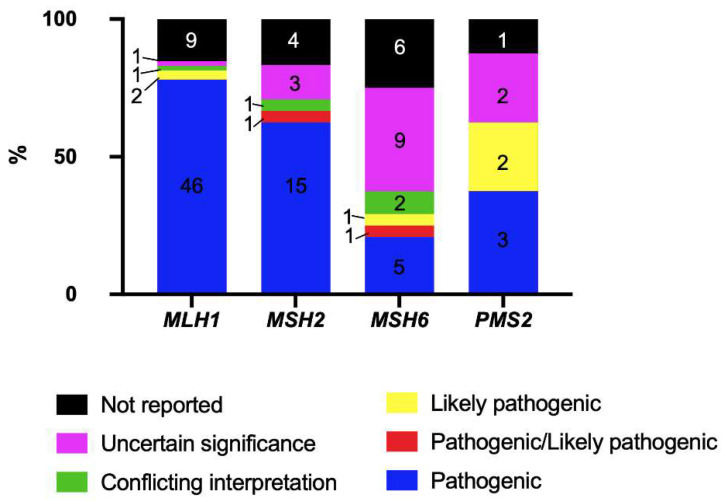
ClinVar classification of MMR variants identified in the cohort. The number in the bars represents the number of variants identified by classification per gene.

According to the ClinVar classification of the variants identified in the MMR genes, a total of 59 variants were obtained in *MLH1*, most of which were pathogenic (46 variants) followed by variants that were not reported (9), likely pathogenic variants (2), variants with a conflicting interpretation of pathogenicity, and one variant of uncertain significance (VUS). In *MSH2,* 24 variants were reported, where the majority were pathogenic (15), followed by those that were not reported (4), VUS (3), those with conflicting interpretation (1) and pathogenic/likely pathogenic variants (1). In *MSH6*, 21 variants were identified, where the majority were VUS (9), followed by variants that were not reported (6), were pathogenic (5), had conflicting interpretation (2), were pathogenic/likely pathogenic (1), and were likely pathogenic variants (1). In *PMS2,* eight variants were identified: three pathogenic, two likely pathogenic, two VUS, and one that was not reported (Figure 3).

With regard to the PV identified, considering those reported in ClinVar and those not previously reported, a total of 96 (96/146) patients had a PV in one of the MMR genes; of these, 69.79% (67/96) had a first-degree cancer family history; 70.83% (69/96) were women, and 29.17% (29/96) were men. The mean age at diagnosis was 41.21 years with a minimum age of 19 and a maximum of 66 years (SD 10.39). In women, the mean age at diagnosis was 41.76 years, with a minimum age of 20 years and a maximum age of 66 years (SD 10.05). In men, the mean age was 39.89 years (SD 9.48), with a minimum age of 19 years and a maximum of 66 years. Regarding clinical stage, the majority of carriers (24.02%, 23/96) presented at stage IIA, followed by stage IIIB in 11.45% (11/96) of the cases (Table 4).

**Figure 4:**
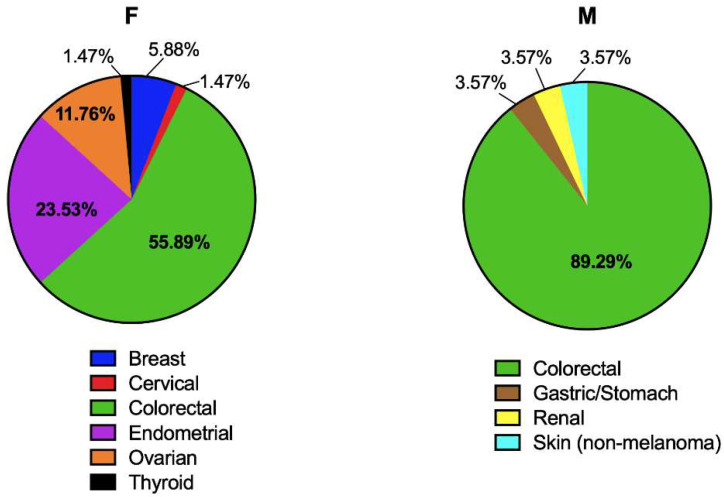
Site of incidence of pathogenic MMR variants identified by gender.

The site of the first cancer diagnosis in PV carriers was, for women and men, colorectal tumors (55.89% and 89.29%, respectively), with a predominance of the adenocarcinoma subtype (Figure 4 and Table 5). In the case of women, the second most common cancer incidence was endometrial cancer, in 23.53% of the cases, all of which were adenocarcinomas. For ovarian cancer (11.76% of the cases), most of them had endometrioid adenocarcinoma histology. Breast cancer represented 5.88% of the PV cases, and they all had invasive ductal carcinoma histology (Figure 4).

**Figure 5:**
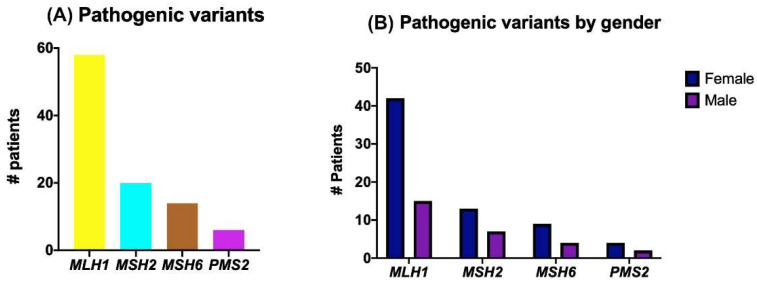
Distribution of pathogenic MMR variants: (**A**) number of pathogenic variants identified by MMR gene; (**B**) pathogenic variants identified by gender.

**Figure 6:**
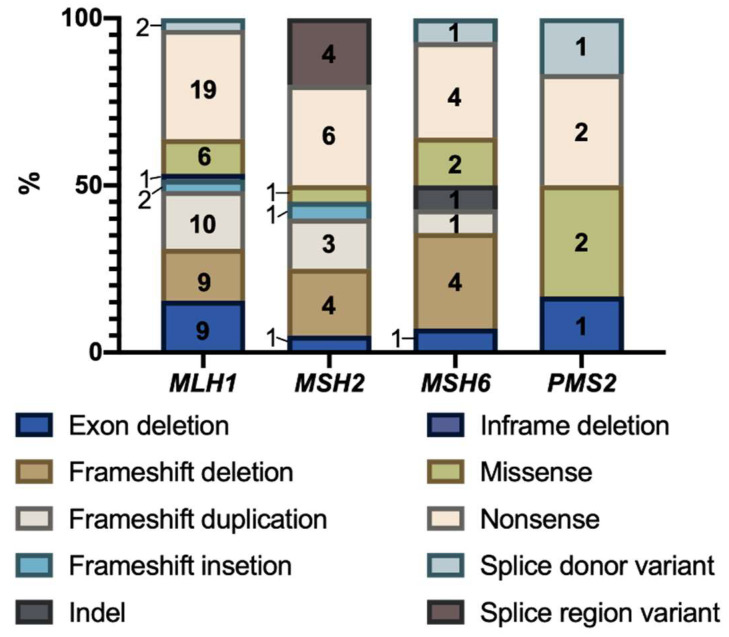
Type of pathogenic variants identified in the cohort of patients carrying a pathogenic variant. The number of variants identified for each type of mutation is indicated inside the bars.

Most of the PVs identified were in *MLH1* (58 PV), and the majority were identified in women (42 women vs. 15 men) (Figure 5). Forty-two of the *MLH1* variants were identified in patients diagnosed with colorectal cancer, eight with endometrial cancer, three with ovarian cancer, two with breast cancer, one with cervical cancer, and one with skin cancer (Table 4). Following this, 20 PVs were identified in *MSH2*, 13 identified in women, and 7 in men (Figure 6). The majority were identified in patients diagnosed with colorectal cancer, followed by endometrial cancer (Table 5). In *MSH6,* 14 PV were identified; 9 identified in women, and 4 in men. Of these, tumor diagnosis corresponded to four PVs in colorectal cancer, four PVs in endometrial cancer, three PVs in ovarian, one PV in breast cancer, and one PV in thyroid cancer. Finally, for *PMS2*, six PVs were identified; four in women and two in men (Figure 5). Three of these were in colorectal cancer, one in breast cancer, and one each in gastric and renal cancer (Table 5). We report four patients with more than one variant in the MMR genes, and seven patients with an MMR gene variant plus a variant in a non-MMR gene, either pathogenic or VUS. It is worth mentioning that there were two patients with a VUS in an MMR gene alongside a pathogenic variant in another gene (Table A1, Appendix A).

The most represented type of PV in the cohort was nonsense mutations, in 31.63% of cases, followed by frameshift deletions (17.35%), frameshift duplications (14.29%), whole exon deletions (12.24%) missense (11.22%), splicing alterations (4.08%), in-frame deletions (1.02%), and indels (1.02%).

In *MLH1*, 19 nonsense mutations, 10 frameshift duplications, 9 duplications, 9 full exon deletions, 6 missense variants, 2 insertions, 2 splice site alterations, and 1 in-frame deletion were reported (Figure 6). Five recurrent variants were found: *MLH1* c.117_306del (del exon 2 and 3) in 5 patients, *MLH1* c.676C > T (p.Arg226Ter) in 13 patients, *MLH1* c.350C > T (p.Thr117Met), *MLH1* c.1852_1854delAAG (p.Lys618del) in 2 patients, and *MLH1* c.2218dupA (p.Ile740Asnfs) in 2 patients. In *MSH2,* six nonsense variants, four alterations in splicing regions, six frameshift deletions, three duplications, and one complete exon deletion, insertion and missense were reported (Figure 5). A recurrent variant was found in three patients: *MSH2* c.942 + 3A > T. In *MSH6*, four nonsense variants, four frameshift deletions, two missense variants and one complete exon deletion, duplication, indel, and splice variant were reported. Finally, in *PMS2,* two nonsense and missense variants, as well as one full exon deletion, and one alteration in the splicing region were reported (Figure 6).

In the case of patients with suspected LS, 30.4% (126/414) had a variant in non-MMR genes (Figure 1), 77.78% of whom (98/126) were women, while 22.22% (28/126) were men. The mean age at diagnosis was 41.36 years, and 12.14% (50/126) had a positive family history. The most representative clinical stage was IV, in 16.12% (20/126) of cases; and 62.90% (78/126) were alive without disease at the end of the study (Table 1). When a variant in non-MMR genes was identified, 61.11% (77/126) corresponded to colorectal cancer, followed by endometrial cancer in 17.46% (22/126), ovarian cancer in 10.32% (13/126), breast cancer in 7.14% (9/126), and gastric and vaginal cancer in 1.59% (2/126 each); in addition, a variant was identified in cervical cancer (Table 2).

**Figure 7:**
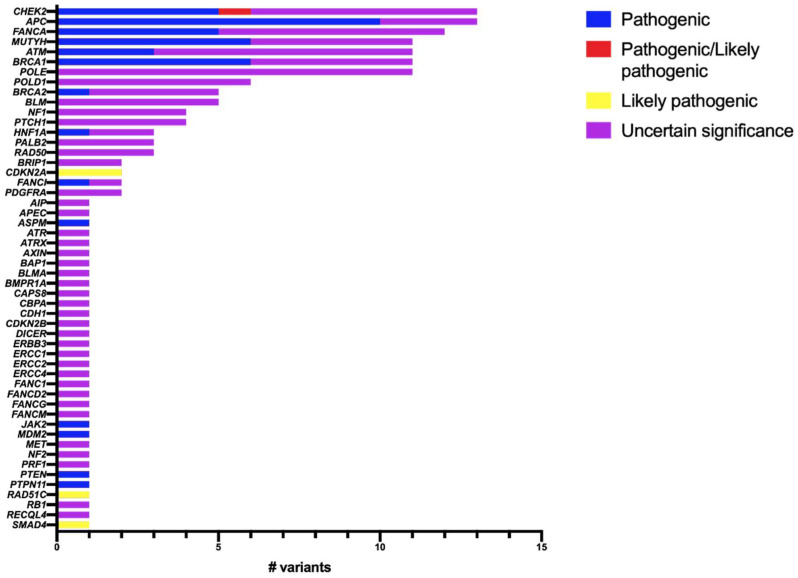
Variants identified in non-MMR genes with their classification. Pathogenic: blue; Pathogenic/likely pathogenic: red; Likely pathogenic: yellow; VUS: purple.

Regarding the variants identified in non-MMR genes, 69.23% (108 variants) were VUS. The genes that presented more than 10 variants were *CHEK2, APC, FANCA, MUTYH, ATM, BRCA1* and *POLE* (Figure 7). We report 23 patients who presented more than one germline variant in different genes. Of these, 12 patients had two VUS, one with two PVs, one with one pathogenic variant with two VUS, and one with one pathogenic variant with three VUS (Table A1, Appendix A).

## 3. Discussion

Through comprehensive hereditary cancer risk assessment, we identified a PV frequency of 28.15% (116/412) in cancer susceptibility genes, in a population of 412 Mexican patients with LS, through NGS. In terms of PVs, 71 were identified in MMR genes, which represents a mutational frequency of 17.23% (71/412).

Regarding the tumor site diagnosis for PV carriers, most of them presented a LS-spectrum cancer, such as colorectal, endometrial, and ovarian cancer. For women, breast cancer was found in fourth place in terms of tumor frequency. The assessment of breast cancer risk for LS is controversial [17,18]. Recent studies have suggested that breast cancer may be included in the cancer risk associated with LS, where a significant increase in breast cancer incidence rates have been found with an earlier age of diagnosis compared to the general population. Nevertheless, other studies show no association and, therefore, do not recommend increased breast cancer surveillance for patients with LS [17,18,19]. This study adds to the evidence to indicate a broader spectrum of associated tumors, where breast cancer represented 5.88% of the MMR pathogenic variant cases, contributing to the comparison and reflection in other populations.

The variants in our studied population were found to be dominated by those identified in *MLH1* (52.67%), followed by *MSH2* (21.42%), *MSH6* (18.75%), and *PMS2* (7.14%). Comparing these results with previous reports in Latin America, our results concur with those of Rossi et al. (2017) and DellaValle et al. (2019), where they report a higher prevalence of *MLH1* at 53.9% and 43%, respectively, followed by *MSH2* at 32.4% and 37%, respectively. Our results differ with those previously reported regarding the percentage of variants found in *MSH6*, where the prevalence cited in the literature ranges from 7 to 9% [13,15,20]; we found a higher percentage of variants in our report. DellaValle identified variants in *MLH1* in 39% of women and 50% of men; *MSH2* in 37% of women and men; *MSH6* in 13% of women and 3% of men; and *PMS2* in 11% of women and 8% of men. In our cohort, a higher percentage of variants was observed in women in all of the genes of the MMR pathway; however, this may be due to the fact that 70% of the cohort were women.

The most frequent type of PV identified was the frameshift type, considering insertions, duplications, and deletions; this was followed by nonsense variants, large deletions, missense variants, and splice-site alterations. These data are comparable with those reported by Vaccaro et al. (2016), who reported that, in their cohort of 98 PVs, frameshift alterations were the most common, followed by nonsense mutations and large deletions; our data differ in the frequency of missense variants, where they report a lower frequency than we identified in our patients.

A recurrent pathogenic variant in *MLH1* was identified in 13 unrelated patients of the cohort. The variant *MLH1* c.676C > T creates a premature termination codon. It was first reported in three siblings from a Turkish family [21]. This variant has been identified in multiple families with LS [22,23,24,25,26], Lynch-like syndrome [27], early onset colorectal cancer [28,29,30], as well as epithelial ovarian cancer [31]. In addition, this variant has been found in the Latin American population in families meeting the Amsterdam and Bethesda criteria in Argentina [13,32], and in one study of the Hispanic population in the United States [33] and Mexico [13].

A variant of uncertain significance (VUS) is defined as a genetic sequence variant, whose association with disease risk is unknown. Since the risk is unknown, it is not clear whether the sequence change is a typical variant, a polymorphism, or represents a disease-causing variant [34]. From the consulted literature regarding Latin America and LS, only two papers report VUS. Vaccaro et al. (2016) report a total of 128 variants, of which, 30 were VUS. Rossi et al. (2017) report a total of 220 variants, of which, 37 were VUS and 1 variant had a conflict of interpretation. In our cohort, we found a lower number of VUS, reporting 15 VUS and 4 variants with conflicting interpretations. However, we have also found a number of VUS in MMR genes, in tumors outside of the LS spectrum. The reporting of a VUS represents a dilemma since it is not known where on the spectrum, from pathogenic to benign, a given VUS falls; carrier status does not stratify family members into those at higher or lower risk. Therefore, it does not provide any benefit in terms of medical management for the carrier or their family [35]. Given this uncertainty, it is worth emphasizing the importance of elucidating the pathogenicity of VUS, in order to provide management and follow-up for both patients and their families.

For the cancer susceptibility genes identified in the cohort that are not involved in LS, we found several genes implicated in the hereditary colorectal cancer landscape: *APC*, *MUTYH*, *POLE*, *POLD1*, and *BLM*, as well as genes resulting in an increased risk of colon cancer as *CHEK2*. The highest number of variants in the cohort was in the *MUTYH* gene, mostly PVs and pathogenic/likely pathogenic variants. Of the 21 *MUTYH* variants identified, 7 were found in patients diagnosed with colorectal cancer. *MUTYH* is a gene whose protein is involved in the base excision repair pathway that detects and protects DNA from oxidative stress [36,37]. The presence of monoallelic variants and medical management of carriers is controversial. Several studies have reported an association with colorectal cancer [37,38], endometrial cancer, and breast cancer [38], sites which were also reported in the present study. In addition, 13 variants, mostly pathogenic, were found in the *APC* gene. Familial adenomatous polyposis (FAP), characterized by the presence of dozens to hundreds of colorectal adenomas, is a hereditary disease caused by germline variants in *APC*, a key tumor suppressor gene in the regulation of the WNT signaling pathway [35,39]. FAP patients have a high risk of developing colorectal cancer, and an increased risk of gastric, small bowel, pancreatic, and thyroid carcinoma, in addition to bone and ophthalmological alterations [39]. In this cohort, 11 of the variants were identified in patients diagnosed with colorectal cancer, but without polyps or other clinical manifestation of FAP. Finally, related to hereditary colorectal cancer, 11 variants in *POLE* and 6 variants in *POLD1* were found. Pathogenic variants in *POLE* and *POLD1* cause PPAP syndrome (polymerase proofreading-associated polyposis), where there is an increased risk of developing colorectal cancer [40]. In the cohort, all variants identified were VUS in colorectal cancer patients.

Different NGS studies have reported that up to 18% of patients under 50 years old, diagnosed with colorectal cancer, have a PV in genes that are not traditionally associated with this neoplasm such as *ATM*, *CHEK2*, and *BRCA1/2* [16]. In the cohort, 13 variants in *CHEK2* were identified, of which, 9 were found in patients diagnosed with colorectal cancer. Most of these variants were of uncertain significance, but five pathogenic variants were identified. *CHEK2* encodes a serine/threonine kinase, activated in response to DNA damage, regulating downstream effector proteins such as p53, BRCA1, and BRCA2. *CHEK2* is a susceptibility gene for several types of cancer such as breast, ovarian, and colorectal cancer, among others [41,42]. In this cohort, 11 variants in *ATM* were identified, most of them diagnosed with colorectal cancer. *ATM* is a master regulatory kinase which is active in response to DNA damage, carriers of heterozygous variants are at risk of breast cancer and, potentially, pancreatic cancer [43]. The most recent version of the NCCN Guidelines for Genetic and Familial CRC Syndromes (version 1.2021), indicates that no evidence has been established; therefore, the risk of colorectal cancer in carriers of *ATM* variants is uncertain.

Likewise, our report showed 11 *BRCA1* variants, 6 PVs and 5 VUS, as well as 9 *BRCA2* variants, 5 PV and 4 VUS. Different studies have found the presence of variants in *BRCA1* and *BRCA2*: in a total of 450 patients with early onset colorectal cancer, four variants were identified in *BRCA1* and one variant in *BRCA2* [44]; in 1260 individuals with a family history of LS, 8% had variants in these genes [45]; and in a cohort of 961 endometrial cancer patients, 1.04% presented with a PV [46]. PVs in *BRCA1* and *BRCA2* are classically associated with hereditary breast and ovarian cancer syndromes [47]. Colorectal cancer is not included in the *BRCA1/2* tumor spectrum; published guidelines do not recommend increased detection of this type of neoplasm in carriers of mutations in these genes. Some studies have suggested a modest link between the risk of developing colorectal cancer and BRCA genes, although the data are often contradictory and cannot establish a causal link between variants and the diagnosis of colorectal cancer in probands [48].

The limitations of this study include: The National Cancer Institute is a countrywide reference oncology hospital, which reflects the population from the central region of Mexico, and the results obtained cannot be generalized to the rest of the country. However, the population of this study is relevant due to the large number of patients analyzed.

Hereditary Cancer Clinic patients must be referred by the attending physician (medical and/or surgical oncologist). If the physician does not suspect a hereditary cancer risk, these patients are not referred and therefore, they are not included. Nowadays, in our Institution there is greater awareness of the importance of cancer risk assessment in the Gynecology and Breast Cancer Units; as a result, our cohort had a higher representation of female patients. The identification of patients with a PV, hence with a new diagnosis of a hereditary cancer syndrome such as LS, benefit from management recommendations for enhanced surveillance, prophylactic surgeries, and cascade testing for cancer prevention [49].

Regarding therapeutic implications, the NCCN guidelines recommend searching for protein loss by immunohistochemistry (IHC) and/or microsatellite instability in all colorectal and endometrial cancer tumors [12]. In our Institute, due to hospital criteria and budget constraints, the pathologist only performs IHC for some cases. Immunotherapy of immune checkpoint inhibition targeting PD1 and CTLA-4 proteins has been shown to be effective in dMMR-MSI-H tumors and has become the standard of care for patients with metastatic colorectal cancer. The FDA has approved the use of the anti-PD1 antibodies pebrolizumab and nivolumab, anti-CTLA4 termelimunab, and the combination of nivolumab and ipilimumab for the treatment of dMMR-MSI-H. It is noteworthy that pMMR-MSI-L tumors demonstrate a lack of response to treatment by immunotherapy, which is attributed to the lower mutational burden of this type of tumor [7,8,9,10]. It is important to know the MSI status of tumors in order to provide appropriate and timely therapeutic options. In addition, we depend on multidisciplinary management so that patients have therapeutic adherence to follow-up and the availability of specific therapies.

To the best of our knowledge, there are no studies of LS that have dealt exclusively with Mexico. There are previous reports on the Mexican population in the Latin American context, with limited LS data [15,16]. This is the first study to report the spectrum of genetic variants in a cohort of patients with LS in the Mexican population, which provides a comprehensive molecular and clinical overview. It is imperative to highlight the importance of identifying carriers of PV in any of the MMR genes or other clinically actionable high-risk genes, who would benefit from either surveillance, targeted therapy, or risk-reducing surgery. The recognition of a population with specific health care needs will make it possible to outline public health strategies and policies.

## 4. Materials and Methods

### 4.1. Patients

This study is a retrospective analysis of a cohort of patients with suspected LS from the Hereditary Cancer Clinic, of the National Cancer Institute, from 2016 to 2021 (Ramirez-Otero et al., 2022, under review). Hereditary Cancer Clinic patients must have been referred by the attending physician (medical and/or surgical oncologist).

Suspected LS patients were selected using the Amsterdam and Bethesda criteria, as well as the NCCN guidelines. For the purposes of describing our population, we grouped patients under the term “suspected Lynch syndrome” for those who met the clinical criteria and underwent genetic testing. Based on molecular analysis results, we further divided them into “MMR”, comprising those patients with pathogenic variants in MMR genes, and therefore with LS; “no variant identified, for those patients with a negative result; and, “non-MMR”, comprising those patients with a variant in a gene other than an MMR gene.

Data such as gender, age at diagnosis, first-degree family history, molecular test result, site of incidence, and subtype of disease were considered.

### 4.2. Molecular Testing

Blood samples were used for molecular testing by next-generation sequencing by an extensive multigene panel, as previously reported (Ramirez-Otero et al., 2022, under review). In brief, 4 milliliters of peripheral blood were obtained from all patients through venipuncture. Genomic DNA was extracted from peripheral blood leukocytes using the commercial Wizard Genomic DNA Purification kit (Promega, Madison, WI, USA). Next generation sequencing (NGS) was performed on the Illumina commercial platform (Illumina, San Diego, CA, USA), using two panels of 263 and 322 genes associated with cancer. The genes analyzed were selected according to their function and association with high, moderate, or low susceptibility to cancer. The patients were assigned to the version panel, according to the date they were enrolled in the study. 

When clinical suspicion was high, multiplex ligation-dependent probe amplification (MLPA; MLPA reagent kit and SALSA MLPA Reagents (MRC-Holland P003, P072) in *MLH1* and *MSH2,* genes was used to identify genomic rearrangements in this MMR genes.

All patients had pre- and post-testing genetic counseling. Informed consent was obtained from each patient. The research protocol was current and was approved by the local research committees.

The variants identified were described using the Genome Variation Society (HGVS) nomenclature guidelines [50]. The variants were described considering the following reference sequences: NM_000249.4 (*MLH1*), NM_000251.3 (*MSH2*), and NM_000179.3 (*MSH6*) y NM_000535.7 (*PMS2*). All identified variants were searched in the ClinVar database.

The variants were classified according to the five-tier class system, as follows: pathogenic, likely pathogenic, uncertain significance, likely benign, and benign variants [51]. Likely benign and benign variants were considered where no variant was identified.

Data were collected in Microsoft Excel, and descriptive statistics such as frequencies and percentages were calculated to describe the characteristics of the population. The mean was calculated with the statistical program R (version 3.6.3). All graphs were made using GraphPad Prism (version 9.0.1).

## Figures and Tables

**Table 1:** Socio-demographic characteristics of the cohort with suspected Lynch syndrome.

	Suspected LS	MMR	No Varaint Identified	Non-MMR Gene
**Total**	412	111	175	126
**Female**	296 (71.84%)	80 (72.07%)	118 (67.43%)	98 (77.78%)
**Male**	116 (28.16%)	31 (27.93%)	57 (32.57%)	28 (22.22%)
**Mean Age at diagnosis**	40.82767	40.63964	40.56	41.36508
**Current age**	44.2718	44.06857	45.18018	44.2619
**Positive family history**	195 (47.33%)	75 (38.46%)	70 (35.89%)	50 (25.64%)
**STATUS**				
**Following with no disease**	258 (62.62%)	80 (72.07%)	101 (57.06%)	78 (62.90%)
**Alive, in treatment**	54 (13.11%)	16 (14.41%)	17 (9.60%)	20 (16.13%)
**Deceased**	56 (13.59%)	10 (9.01%)	30 (16.95)	16 (12.90%)
**Loss to follow-up**	44 (10.68%)	5 (4.5%)	29 (16.38%)	10 (8.06%)

**Table 2:** Type of diagnosed cancer in patients with suspected Lynch syndrome, with MMR variants, no variant identified, and with variants in other genes.

	Suspected LS	MMR	No Variant Identified	Non-MMR Gene
**Total**	412	111	175	126
**Colorectal**	240 (58.25%)	65 (58.56%)	98 (56%)	77 (61.11%)
**Endometrial**	78 (18.93%)	20 (18.02%)	36 (20.57%)	22 (17.46%)
**Ovarian**	43 (10.44%)	12 (10.81%)	18 (10.29%)	13 (10.32%)
**Breast**	26 (6.31%)	5 (4.50%)	12 (6.86%)	9 (7.14%)
**Gastric/Stomach**	10 (2.43%)	2 (1.80%)	6 (3.43%)	2 (1.59%)
**Cervical**	3 (0.73%)	1 (0.9%)	1 (0.57%)	1 (0.79%)
**Renal**	1 (0.24%)	1 (0.9%)	0	0
**Skin (non-melanoma)**	1 (0.24%)	1 (0.9%)	0	0
**Thyroid**	1 (0.24%)	1 (0.9%)	0	0
**Appendiceal cancer**	1 (0.24%)	0	1 (0.57%)	0
**Brain**	1 (0.24%)	0	1 (0.57%)	0
**Other**	2 (0.49%)	2 (1.8%)	0	0
**Pancreatic**	2 (0.49%)	1 (0.9%)	1 (0.57%)	0
**Prostate**	1 (0.24%)	0	1 (0.57%)	0
**Vaginal**	2 (0.49%)	0	0	2 (1.59%)

**Table 3:** Histological subtype by cancer type in patients with suspected Lynch syndrome, with MMR variant carriers, cases of no variant identified, and carriers of variants in non-MMR genes.

		Suspected LS	MMR	No Variant Identified	Non-MMR Gene
**Colorectal**	Adenocarcinoma	229	62	93	74
	Mucinous cystadenocarcinoma	4	0	3	1
	Poorly differentiated	7	3	2	2
**Endometrial**	Adenocarcinoma	20	7	7	6
	Clear cell carcinoma	1	1	0	0
	Endometrioid adenocarcinomas	53	12	27	14
	Serous adenocarcinomas	1	0	0	1
	Unknown	1	0	1	0
	Other	2	0	1	1
**Ovarian**	Adenocarcinoma	2	1	0	1
	Clear cell carcinoma	4	0	3	1
	Dysgerminoma	1	1	0	0
	Endometrioid adenocarcinomas	16	5	6	5
	Mucinous cystadenocarcinoma	7	1	3	3
	Other	1	0	1	0
	Papillary serous carcinoma	4	1	1	2
	Poorly differentiated	1	1	0	0
	Serous adenocarcinomas	6	2	3	1
	Serous cystadenocarcinoma	1	0	1	0
**Breast**	Ductal carcinoma—invasive	21	5	9	7
	Mucinous cystadenocarcinoma	2	0	1	1
	Other	3	0	2	1
**Gastric/** **Stomach**	Adenocarcinoma	9	2	6	1
	Poorly differentiated	1	0	0	1
**Renal**	Unknown	1	1	0	0

**Table 4:** Socio-demographic and histopathological characteristics of the cohort of patients with pathogenic variants (PV) in one of the MMR genes.

	Number of Cases (%)
**Female**	68 (70.86%)
**Male**	28 (29.17%)
**Diagnostic age**	41.21 (19–66; SD 10.39)
**Family history**	67 (69.79%)
**Primary Tumor**	
**Colorectal**	63 (65.62%)
**Endometrial**	16 (16.66%)
**Ovarian**	8 (8.33%)
**Breast**	4 (4.16%)
**Cervical**	1 (1.04%)
**Gastric/Stomach**	1 (1.04%)
**Renal**	1 (1.04%)
**Thyroid**	1 (1.04%)
**Skin (non-melanoma)**	1 (1.04%)
**TNM Staging**	
**I**	1 (1.04%)
**IA**	8 (8.33%)
**IC**	3 (3.12%)
**II**	6 (6.25%)
**IIA**	23 (24.02%)
**IIB**	4 (4.16%)
**IIC**	1 (1.04%)
**III**	2 (2.08%)
**IIIA**	3 (3.12%)
**IIIB**	11 (11.45%)
**IIIC**	9 (9.37%)
**IV**	6 (6.25%)
**IVA**	2 (2.08%)
**IVB**	2 (2.08%)
**LMA**	2 (2.08%)
**N/A**	13 (13.54%)

**Table 5:** Type of cancer and histological subtype in patients with pathogenic MMR variants and number of patients with variants by MMR gene.

Disease Type	# Patients Number	*MLH1*	*MSH2*	*MSH6*	*PMS2*	Disease Subtype
**Breast**	4	2	0	1	1	Ductal carcinoma—invasive
**Cervical**	1	1	0	0	0	Endovercival adenocarcinoma
**Colorectal**	63	42	14	4	3	Adenocarcinoma
						Poorly differentiated
**Endometrial**	16	8	4	4	0	Adenocarcinoma
						Clear cell carcinoma
						Endometrioid adenocarcinomas
**Gastric/** **Stomach**	1	0	0	0	1	Adenocarcinoma
**Ovarian**	8	3	2	3	0	Adenocarcinoma
						Endometrioid adenocarcinomas
						Papillary serous carcinoma
						Poorly differentiated
						Serous adenocarcinomas
**Renal**	1	0	0	0	1	Unknown
**Skin(non-melanoma)**	1	1	0	0	0	Other
**Thyroid**	1	0	0	1	0	Papillary

## Data Availability

The data presented in this study are openly available in FigShare at 10.6084/m9.figshare.20404746.

## Supplementary Materials

The following supporting information can be downloaded at: https://www.mdpi.com/article/10.3390/ijms231911549/s1.

## Appendix A

**Table A1:** Other genes.

PMMR Genes	
**P *MLH1* c.676C > T + LP *PMS2* c.1A > G**	P *BRCA2* + VUS *PTCH1*
**P *MSH6* c.3838C > T + NR *MSH6* c.1495delA**	VUS *BRCA1* + VUS *BRCA1*
**P *MLH1* c.350C > T + VUS MSH6 + VUS MSH6**	VUS *NF1* + VUS *NF2*
**P *MLH1* C.2092_2093delTC + VUS *MSH6* + P *CHEK2* c.279G > A**	P *MUTYH* + P *CHEK2*
**VUS *MSH6* + P *MUTYH***	VUS *FANCA* + VUS *CHEK2*
**VUS *PMS2* + VUS *RAD50* + P *CHEK2***	P *CHEK2* + VUS *BLM*
**P *MLH1* c.1459C > T + P *BRCA2***	P *HNF1A* + VUS *POLD1*
**P *PMS2* c.485T > A + P *BRCA2* c.9235delG**	VUS *ATM* + VUS *POLE*
**P *PMS2* del7-11 + *FACD2* + VUS *FANCI***	P *BRCA2* + VUS *BRCA1*
**P *MLH1* c.2218dupA + P *MUTYH* c.1145G > A**	P *FANCI* + VUS *RAD50*
**P *MLH1* del1-2 + P *FANCA***	VUS *FANCI* + VUS *POLE*
**P *MLH1* c.676C > T + P/LP *CHEK2* c.1237delT**	VUS *ERCC1* + VUS *POLE*
**P *MSH2* c.942 + 3A > T + P *BRCA2* c.9699_9702del**	VUS *POLD1* + VUS *ERCC4*
**VUS *PMS2* + P *CHEK2* + *VUS* RAD50**	VUS *ERCC4* + VUS *FACG*
	VUS *BLM* + VUS *FANCA*
	VUS *BLM* + VUS *PDGFRA*
	VUS *BRIP1* + VUS *CDKN2B*
	VUS *CBPA* + VUS *MET*
	P *ATM* + VUS *PTCH1* + VUS *CHEK2*
	VUS *BMPR1A* + VUS *FANCA* + VUS *ATM*
	VUS *APEC* + VUS *POLD1* + VUS *BRCA1*
	VUS *APC* + VUS *POLE* + VUS *POLD1*
	P *CHEK2* + VUS *BRCA1* + VUS *FANCA* + VUS *FANCD2*
