# Supplementary material for: A Previously Unrecognized Molecular Landscape of Lynch Syndrome in the Mexican Population
Source: Int J Mol Sci. 2022 Sep 30;23(19):11549. doi: 10.3390/ijms231911549 (PMC9569652; doi:10.3390/ijms231911549)
Supplement: Supplementary file 1 [file ijms-23-11549-s001.zip › ijms-1869836-supplementary.pdf]

Table S1. TNM staging by histopathological subtypes of each type of cancer diagnosed in the cohort of patients with Suspected Lynch syndrome. The numbers, represent the total number of patients of each caner type and histological subtype. AML: locally advanced disease, N/A: not available.

Cancer Type	Histological subtype	TNM Staging	Suspected LS (412)	MMR (113)	No variant (177)	Non-MMR (122)
Colorectal (240)	Adenocarcinoma (229)	I	2			2
		IA	5	2	2	1
		IC	1	1		
		II	5	2	3	
		IIA	38	18	13	7
		IIB	8	2	3	3
		IIC	7	2	3	2
		III	4	2		2
		IIIA	9	1	4	4
		IIIB	28	12	7	4
		IIIC	14	5	5	4
		IV	46	2	27	17
		IVA	9	2	4	3
		IVB	2	1		1
		IVC	2	1		1
		In situ	2		1	1
		LMA	14	1	6	7
		N/A	33	9	15	9
	Mucinous cystadenocarcinoma (4)	IIA	2		2	
		IV	2		1	1
	Poorly differentiated (7)	IIA	1			1
		IIB	1	1		
		IIIC	1			1
		IV	1		1	
		LMA	1	1		
		N/A	2	1	1	
Endometrial (78)	Adenocarcinoma (20)	IA	5	2	2	1
		II	3	1		2
		IIIA	3	1	1	1
		IIIB	1		1	
		IIIC	6	2	2	2
		IVB	2	1	1	
	Clear Cell (1)	N/A	1	1		
	Endometrioid adenocarcinoma (53)	I	1		1	
		IA	20	3	10	7
		IB	3		3	

		II	5	3	2	
		IIA	3	2		
		IIIA	7	1	4	2
		IIIB	1			1
		IIIC	7	3	3	1
		IV	1		1	
		IVA	1			1
		IVB	3		3	
		N/A	1			1
	Serous adenocarcinoma (1)	IIIC	1			1
	Other (2)	IA	1			1
		IV	1		1	
	Unknown (1)	In situ	1		1	
Ovarian (43)	Adenocarcinoma (2)	IV	1			1
		IVB	1	1		
	Clear cell Carcinoma (4)	IA	1		1	
		IC	1		1	
		IIIB	1		1	
		IVB	1			1
	Dysgerminoma (1)	IIIB	1	1		
	Endometriod adenocarcinoma (16)	IA	5	2	1	2
		IC	3	1	1	1
		IIA	1		1	
		IIIA	1		1	
		IIIC	3		3	
		IV	1	1		
		N/A	2	1	1	
	Mucinous cystadenocarcinoma (7)	IA	1		1	
		IC	1			1
		IIIC	1			1
		N/A	4	1	2	1
	Papillary serous carcinoma (4)	IC	1			1
		IIIC	3	1	1	1
	Poorly differentiated (1)	IVB	1	1		
	Serous adenocarcinoma (6)	IA	3	1	2	
		IC	1	1		
		IIIA	1		1	
		N/A	1			1
	Serous Custadenocarcinoma (1)	IV	1		1	
	Other (1)	IB	1		1	
Breast (26)	Ductal Carcinoma-invasive (21)	IA	3	1	2	
		IIA	4	2	1	1

		IIB	4	1	1	2
		IIIA	4	1	2	1
		IIIB	1		1	
		IIIC	1			1
		IV	1			1
		In situ	1			1
		N/A	2		2	
	Mucinous Cystadenocarcinoma (2)	IIA	1			1
		IIIA	1		1	
	Other (3)	IA	1		1	
		II	1			1
		IIIB	1		1	
Gastric/Stomach (10)	Adenocarcinoma (9)	IIIA	2		2	
		IIIC	1		1	
		IV	4	2	2	
		LMA	1			1
		N/A	1			1
	Poorly differentiated (1)	N/A	1			1
Cervical (3)	Adenocarcinoma (2)	In situ	1			1
		N/A	1		1	
	Endocercival adenocarcinoma (1)	IIA	1	1		
Pancreatic (2)	Adenocarcinoma (1)	IIA	1		1	
	Other (1)	IV	1	1		
Renal (1)	Unknown (1)	I	1	1		
Appendiceal cáncer (1)	Mucinous cystadenocarcinoma (1)	IV	1		1	
Brain (1)	Other (1)	N/A	1		1	
Prostate (1)	Adenocarcinoma (1)	N/A	1		1	
Skin (non-melanoma) (1)	Other (1)	N/A	1	1		
Thyroid (1)	Papillary (1)	IIA	1	1		
Vaginal Cancer (2)	Adenocarcinoma (1)	IIIC	1			1
	Basal cell carcinoma (1)	IB	1			1
Other (2)	Adenocarcinoma	IIIA	1	1		
	Other	IIIB	1	1		
